# Wildlife Mortality Investigation and Disease Research: Contributions of the USGS National Wildlife Health Center to Endangered Species Management and Recovery

**DOI:** 10.1007/s10393-013-0897-4

**Published:** 2014-01-14

**Authors:** Christopher J. Brand

**Affiliations:** U.S. Geological Survey, National Wildlife Health Center, 6006 Schroeder Rd., Madison, WI 53711 USA

**Keywords:** endangered species, disease, mortality, management, recovery, emerging diseases, diagnostic, vaccination

## Abstract

The U.S. Geological Survey—National Wildlife Health Center (NWHC) provides diagnostic services, technical assistance, applied research, and training to federal, state, territorial, and local government agencies and Native American tribes on wildlife diseases and wildlife health issues throughout the United States and its territories, commonwealth, and freely associated states. Since 1975, >16,000 carcasses and specimens from vertebrate species listed under the Endangered Species Act have been submitted to NWHC for determination of causes of morbidity or mortality or assessment of health/disease status. Results from diagnostic investigations, analyses of the diagnostic database, technical assistance and consultation, field investigation of epizootics, and wildlife disease research by NWHC wildlife disease specialists have contributed importantly to the management and recovery of listed species.

## Introduction

Knowledge of morbidity and mortality factors for endangered species and their relative importance and impact on endangered populations is a critical component of understanding the driving forces of species declines and potential for extinction, as well as identifying limiting factors to species recovery. Wilson (1992) identified the major drivers of species imperilment, including: habitat destruction, alien species, pollution, overexploitation, and disease. However, few quantitative studies have been conducted on the roles of factors that threaten species (Wilcove et al. 1998). Infectious diseases have historically seldom been considered to be potential drivers of the decline of wildlife species to the point of endangerment or extinction (MacPhee and Greenwood 2013). In an examination of the 2004 IUCN Red List of Threatened Species, Smith et al. (2006) found only 25 (<4%) of 724 plant and animal species extinctions since 1900 were linked to infectious diseases; and of 2,852 critically endangered species, 223 (<8%) have infectious disease listed as a contributing factor to their endangered status. However, few of these cases were sufficiently studied or documented, and there was little conclusive evidence to support that disease was in fact a contributing factor (Smith et al. 2006). Among the few early studies indicating infectious disease as a primary factor in the decimation of species was Warner’s (1968) treatise on the role of introduced avian malaria (*Plasmodium relictum*) and possibly avian pox in the extinction of 16 endemic Hawai’ian forest birds and declines to endangered status of numerous others.

More recently, disease has been increasingly recognized as a major factor in species population declines and extinction as well as being an impediment to recovery of endangered species. Examples include: sylvatic plague (*Yersinia pestis*; Jachowski and Lockhart 2009), canine distemper virus in black-footed ferrets (*Mustela nigripes*; Williams et al. 1988), chytridiomycosis in several amphibian species (Schloegel et al. 2006; Skerratt et al. 2007; Daszak et al. 1999), transmissible facial tumor disease in the Tasmanian devil (*Sarcophilus harrisii*; McCallum et al. 2009). Pedersen et al. (2007) list 31 pathogens causing population declines or reduced fitness in mammals on the 2006 IUCN Red List of Threatened Species. Newly discovered and emerging diseases in wildlife have accounted for many of these cases. Driving forces associated with the exacerbation of the occurrence and impacts of such emerging diseases include introduction of exotic species and their pathogens, pathogen introduction from expanding world commerce and travel, large scale habitat and land-use changes in quality, quantity, and distribution; and climate change. As the impact of disease on imperiled species is being increasingly recognized, so is the need to identify their causes, quantify their impact and demographic role, and develop solutions to aid in preventing their extinction and hastening their recovery.

This paper presents examples and cases of contributions made by the U.S. Geological Survey (USGS) National Wildlife Health Center (NWHC) to the survival and recovery of endangered species through identification and quantification of morbidity and mortality factors, technical assistance on wildlife disease-related issues, field investigation of epizootics, and through research and development to assist wildlife and natural resource managers in disease identification, prevention, and management in endangered species.

## The USGS National Wildlife Health Center

The NWHC is a federal science center established in 1975 under the U.S. Fish and Wildlife Service (USFWS), and later transferred to the USGS. With a mission to “…provide national leadership to safeguard wildlife and ecosystem health through dynamic partnerships and exceptional science,” the NWHC provides diagnostic services, technical assistance, applied research, and training to federal, state, territorial, and local government agencies and Native American tribes on wildlife diseases and wildlife health issues throughout the United States and its territories, commonwealth, and freely associated states. Diagnostic and research laboratories and experimental facilities are located in Madison, Wisconsin, with a Honolulu Field Station (HFS) in Honolulu, Hawai’i, to address specific disease issues in Hawai’i and Pacific islands (see www.nwhc.usgs.gov).

## Diagnostic Database Supports Management and Regulatory Decisions

Since its establishment in 1975, the NWHC has conducted necropsies and laboratory studies to determine causes of morbidity, mortality, or health status in >110,000 wildlife specimens submitted for diagnosis. Of these, >16,000 were species that are, or were formerly, listed under the U.S. Endangered Species Act in the categories of: candidate, threatened, endangered, or delisted in all or part of their range; or listed by the International Union for the Conservancy of Nature (IUCN), or Convention on the International Trade in Endangered Species (CITES). These submissions represented 136 species from all classes of vertebrates (birds 78%, reptiles 11%, mammals 10%. amphibians 2%, fish <1%). The NWHC Diagnostic Database is comprised of information and data from each of the >110,000 carcasses or specimens submitted. The database has been used extensively to provide summaries and analyses of status and trends of mortality factors for use in wildlife management and regulatory decisions, including those that involve or affect endangered species. For example, since 1975, >1,200 bald eagle (*Haliaeetus leucocephalus*) carcasses have been examined at NWHC. Cumulative data from bald eagles have been analyzed periodically and used in major decision-making processes:

### Non-toxic Shot Regulations for Waterfowl Hunting

Lead poisoning in birds from ingesting spent lead shotgun pellets has been known as a major mortality factor in waterfowl since the 1870s (Grinnell 1894). Despite this, it wasn’t until the 1970–1980s that significant pressure mounted to require non-toxic shot for waterfowl hunting (USFWS 1976, USFWS 1986, Friend et al. 2009). Part of the impetus for this conversion involved a suit brought about by the National Wildlife Federation to ban the use of lead shot based in part on the increasing number of secondary lead poisoning cases in bald eagles from consuming crippled and lead poisoned waterfowl. In an analysis of 1,429 bald eagles necropsied by the NWHC and Patuxent Wildlife Research Center (at that time part of the USFWS) lead poisoning accounted for 52% of poisoned eagles (USFWS 1986). Later reports from NWHC (Franson et al. 1995; NWHC unpublished) added significantly to these data and were a major contribution to legislation calling for the phase-in and eventual conversion from lead shot to non-toxic shot for waterfowl hunting in the United States in 1991.

### Powerline Electrocution

NWHC diagnostic data from bald and golden (*Aquila chrysaetos*) eagles were instrumental in identifying the extent of electrocution and traumatic deaths associated with powerlines. Franson et al. (1995) reported that, of 4,300 eagles examined during a 30-year period between the 1960s and 1990s, 12% of bald eagles and 25% of golden eagles died from electrocution, and Thomas (1999) reported numbers of eagle deaths from electrocution during 1975–1997 by state, with the majority of cases occurring in Western states, Alaska, and Florida. These results contributed to cooperative establishment of guidelines by the Edison Electric Institute’s Avian Power Line Interaction Committee (APLIC) and the USFWS in the construction and retrofitting design of powerlines to reduce this cause of mortality in eagles and other raptors (APLIC and USFWS 2005).

### Pesticide Poisoning

Cumulative data on bald eagle mortality factors also revealed a relatively high proportion of deaths caused by agricultural pesticides. Franson et al. (1995) reported 139 eagles in 25 states dying from organophosphate (OP) compound and carbamate poisoning. These data, along with data from other wildlife mortality from these pesticides, were used to support restrictions on the use of some OPs and carbamates, including removal of diazinon from use for turf applications and limitations on the use of carbofuran (Glaser 1995).

## Support for Wildlife Law Enforcement

Diagnostic examinations are important not only in determining the cause of death (COD) of victims, but also in determining contributing factors that may have led to the COD as well as documenting factors that were *not* contributing to mortality. Such wildlife necropsies require a comprehensive approach to diagnostic examination supported by an interdisciplinary team of laboratory and pathology support, and prove invaluable when used to support cases of wildlife mortality that involve violation of wildlife laws. The NWHC provides diagnostic support for federal wildlife law enforcement officials in identifying potential cases to pursue and providing legal-quality evidence. A total of 963 carcasses or specimens submitted from listed species were analyzed in support of law enforcement officials, primarily from the USFWS (NWHC unpublished data), largely involving illegal take of Federally protected wildlife under Endangered Species Act, Migratory Bird Treaty Act, Bald and Golden Eagle Protection Act, and Marine Mammal Protection Act.

## Field Investigation of Epizootics in Endangered Species

Field investigations of mortality factors in endangered species, integrated with demographic and ecological studies, can provide important information on the relative importance of various mortality factors and impact on endangered populations that can be used to guide management decisions. The Laysan duck (*Anas laysanensis*), once widely distributed along the Hawai’ian archipelago, has been restricted for the past 150 years to a single population numbering 400–500 birds on Laysan Island (Moulton and Weller 1984; Reynolds and Work 2005). Concern over the risk for extinction of this species led to the translocation of a portion of the population to Midway Atoll in 2004 and 2005 (Reynolds et al. 2008). In November 1993, the occurrence of an unusually large number of Laysan duck carcasses on Laysan Island in the Northwestern Hawai’ian Islands prompted a field investigation of the event by the NWHC HFS. Emaciation complicated by parasitic infection by *Echinuria uncinata* was diagnosed as the cause of death, and recommendations were made for anthelmintic treatment of infected ducks, particularly those to be translocated in the future (Work et al. 2004). During 1998–2003, in preparation for the translocation, a collaborative study between the USGS Pacific Islands Ecosystems Research Center and the HFS determined mortality rates, body condition, and causes of mortality of Laysan ducks through intensive mark-resighting and recovery of 297 ducks and diagnostic necropsies of carcasses (Reynolds and Work 2005). This study attributed a high duckling mortality (0.7–0.9) to habitat-associated overcrowding and brood separation, leading to duckling trauma and exposure-related factors as the major causes of death. Reynolds and Work (2005) provided further habitat management recommendations for this population, as well as information on managing potential habitat-limiting factors that could affect the translocated population. During 2004–2005, following extensive habitat management on Midway, 42 ducks that were treated with ivermectin and tested negative for *E. uncinata* were translocated to Midway Atoll. Since then, this parasite has not been detected in Laysan ducks on Midway. By 2007, Laysan ducks translocated to Midway Atoll increased to an estimated population >200 (Reynolds et al. 2008). However, in August 2008, an epizootic was discovered on Midway, prompting a field investigation. Avian botulism (*Clostridium botulinum* type C toxin) was diagnosed as the cause of mortality that killed at least 181 adult, fledgling and duckling Laysan ducks (Work et al. 2010). The epidemiological investigation identified the likely site and source of the botulinum toxin as a concrete catchment basin and documented the geographic expansion of mortality to 12 wetlands concurrent with peak dispersal of fledglings on the islands of Midway. Based on this investigation, recommended management options undertaken by the USFWS to control and prevent avian botulism included annual flooding of the concrete catchment basin in summer; draining, sludge removal, and cleaning of the basin during fall and winter months; vegetation removal around wetlands to facilitate carcass detection; and increased frequency of searches to detect sick and dead birds, especially during summer months (Reynolds et al. 2011). The effect of these management actions on reducing mortality from avian botulism cannot be directly determined. Botulism recurs on Midway, but not at the magnitude of the outbreak in 2008. However, Laysan ducks on Midway increased to >400 in 2010.

## Development of Vaccines for Endangered Species

The use of commercial vaccines to prevent disease in free-living endangered species has received increasing attention in programs, for example, to prevent rabies in Ethiopian wolves (*Canis simensis*; Randall et al. 2004; Stewart 2009), West Nile virus in California condors (*Gymnogyps californianus*; Chang et al. 2007), and a measles-like respiratory disease in mountain gorillas (*Gorilla beringei beringei*; Ryan and Walsh 2011). Advances in recombinant DNA technology and other new vaccine technologies have expanded the opportunities to develop safer and more efficacious vaccines specific for wildlife species and suitable for mass distribution to free-living wildlife (Rupprecht et al. 2004). The NWHC has developed and tested vaccines that have been used in management plans for several endangered species. In a collaborative study with USGS Patuxent Wildlife Research Center (PWRC), Olsen et al. (2009) studied the serological response of sandhill cranes (*Grus canadensis*) to vaccination against West Nile virus (WNV) using a commercial equine inactivated vaccine. Vaccinated sandhill cranes responded with higher titers to WNV and had viremias for shorter periods of time than controls; unvaccinated cranes also had lesions in the brain and brain stem after challenge, whereas vaccinated cranes did not (Olsen et al. 2009). While this vaccine was not tested specifically in whooping cranes (*G. americana*), demonstration of its protection against WNV in surrogate sandhill cranes resulted in the decision to vaccinate whooping cranes raised at PWRC and at the International Crane Foundation (ICF), Baraboo, Wisconsin, as part of their health checks and preparation for reintroduction to establish a new Florida population. Whooping cranes are also vaccinated against eastern equine encephalitis prior to reintroduction as a result of separate studies at PWRC using an inactivated human vaccine against Eastern Equine Encephalitis (Carpenter et al. 1992; Olsen et al. 1997).

### Vaccination of Black-Footed Ferrets and Prairie Dogs Against Plague

Black-footed ferrets and prairie dogs (*Cynomys* spp.) are highly susceptible to sylvatic plague (*Yersinia pestis*), and this disease is considered one of the major impediments to recovery of the endangered black-footed ferret (Jachowski and Lockhart 2009) as well as the threatened Utah prairie dog (*C. parvidens*; USFWS 2009). Antolin et al. (2002) provide a synopsis of the plight of black-footed ferrets and the role and impact of plague on ferrets and prairie dogs, and Abbott et al. (2012) summarize NWHC’s role in the development and testing of two vaccines against plague, one for ferrets and one for prairie dogs, that are currently being deployed. To protect captive-born black-footed ferrets from plague upon release, the NWHC tested an injectable protein vaccine (F1 and V antigens of *Y. pestis*), developed by the U.S. Army Medical Research Institute of Infectious Disease (Powell et al. 2005) in experimental studies. Vaccinated ferrets developed significant antibody titers against F1 and V which lasted more than 3 years, and survived challenge with virulent *Y. pestis* by both subcutaneous injection and oral routes (Rocke et al. 2008). This F1/V vaccine is now being administered to all captive-born black-footed ferrets prior to release as well as to wild-caught ferrets at some locations. In a study in Montana, Matchett et al. (2010) and Biggins et al. (2010) demonstrated that survival of released ferrets that were vaccinated against plague was significantly greater than that of unvaccinated ferrets that were released. The F1/V vaccine has also been deployed to protect free-living ferrets in the face of a plague outbreak in prairie dogs in the Conata Basin of South Dakota (Livieri 2013).

While vaccination with the F1/V vaccine may prevent plague in individual ferrets, prairie dogs remain subject to plague epizootics that can cause >90% mortality, thus almost eliminating the ferret’s prey base. To prevent plague in prairie dogs, the NWHC, in partnership with the University of Wisconsin School of Veterinary Medicine, developed and tested (for efficacy, safety, duration of immunity, reproduction effects and efficacy, and safety in non-target species) a recombinant vaccine against plague using a raccoon pox-vector (RCN) to express the F1 and Vt antigens (Rocke et al. 2010). The RCN-F1-Vt vaccine has been incorporated into an oral bait matrix that is highly palatable to prairie dogs, along with rhodamine B as a biomarker for animals consuming the bait. Initial field trials of this vaccine in 2012 to determine safety in the field and rates of bait consumption and immune response to vaccination demonstrated a high rate of bait uptake within the first 48 h (Rocke, personal communication). Further field trials will continue in 2013 in prairie dog colonies in 8 western states. If successful, oral vaccination of prairie dogs against plague will be an additional tool that can be used to enhance the recovery of the black-footed ferret as well as the threatened Utah prairie dog.

## Disease and Health Considerations in Captive Breeding, Translocations, and Reintroduction of Endangered Species

Breed et al. (2009) discuss the importance of disease considerations in the captivity, translocation, and reintroduction of endangered wildlife. Input of wildlife disease specialists is an important part of this process; as well as active health and disease monitoring, including postmortem examinations. NWHC staff have been involved in endangered species recovery teams and their working groups in the planning and risk assessment, disease monitoring, and clinical examinations for numerous endangered species recovery programs involving captive breeding, translocation and reintroduction, such as for the masked bobwhite (*Colinus virginianus ridgwayi*), black-footed ferret, Puerto Rican parrot (*Amazona vittata*), whooping crane, néné (*Branta sandvicensis*), Nihoa honeycreeper (*Telespyza ultima*), palila (*Loxioides bailleui*), and Laysan duck.

## Pathogen Discovery: New Diseases Identified in Endangered Species

The International Crane Foundation (ICF) is a non-governmental conservation organization located in Baraboo, Wisconsin, dedicated to conservation of the world’s cranes and their habitats. The ICF partners with federal, state and international governments in promoting captive breeding and release of endangered cranes, and maintains breeding pairs of all crane species. In March 1978, a mortality event occurred at ICF resulting in the deaths of 18 of 51 cranes of 14 species. Of the 18 birds that died, 7 were threatened or endangered (5 Japanese cranes [*Grus japonicus*] and 2 hooded cranes [*G. monachus*]). Carcasses were submitted to NWHC, where a previously-unknown herpesvirus, later named inclusion body disease of cranes (IBDC), was isolated and determined to be the cause of mortality (Docherty and Henning 1980). The NWHC participated with ICF in development of on-site management actions and facility design to control this new disease, conducted additional follow-up studies (Docherty and Romaine 1983; Shuh et al. 1986), and recommended further preventive actions and national surveillance for IBDC in captive cranes (Dein and Docherty 1988). While national surveillance of captive cranes has not been adopted, serological testing of all cranes at the ICF is conducted annually to monitor for its recurrence. Further mortality from IBDC has not occurred at ICF, nor in North America. The source of the herpesvirus was not identified, but likely was introduced by recent international importation of several crane species.

In winter 1994–1995, 29 bald eagles were found dead at De Gray Lake, Arkansas. In 1996–1997, an additional 26 dead bald eagles, as well as American coots (*Fulica americana*), were found at the same lake and nearby Lake Oachita. These deaths represented 30–65% of the overwintering bald eagle population at these sites. Eagle carcasses (5 from 1994–1995 and 19 from 1996–1997) were submitted to the NWHC by the Arkansas Game and Fish Commission. Extensive diagnostic testing and histopathology led to the diagnosis of a previously undescribed syndrome, termed avian vacuolar myelinopathy (AVM) (Thomas et al. 1998). Syndromic surveillance was initiated across the southeastern US, and by 1999 AVM was identified in bald eagles, waterfowl, and several other avian species in North Carolina and South Carolina, continues to occur sporadically at these locations, and has now been documented in Georgia and Texas (Fischer et al. 2002). While the specific etiology causing AVM is still to be confirmed, field and laboratory studies by the NWHC (Rocke et al. 2005), the Southeastern Cooperative Wildlife Diseases Study and others (Birrenkott et al. 2004; Wilde et al. 2005) have implicated a biotoxin produced by algae associated with the exotic aquatic plant *Hydrilla verticillata*.

White-nose syndrome (WNS) in bats, caused by a newly discovered fungal pathogen, *Pseudogymnoascus* (formerly *Geomyces*) *destructans*, has emerged as one of the most devastating wildlife diseases in North America. This disease has rapidly spread from its original site of discovery in a cave in New York in 2006 to now include 22 states and five Canadian provinces (www.whitenosesyndrome.org/about/where-is-it-now) where it has killed an estimated 5.7–6.7 million cave-dwelling bats during hibernation (Froschauer 2012). This cold-loving fungus was first isolated at NWHC, where Koch’s postulates were fulfilled and its pathogenesis described (Blehert et al. 2009; Cryan et al. 2010; Lorch et al. 2011; Meteyer et al. 2012). Its impact on the once-common little brown bat (*Myotis lucifugus*) in Northeastern U.S. has been the greatest, where Frick et al. (2010) suggest this species may now face regional extinction. Additionally, WNS has been diagnosed at NWHC in endangered Indiana (*M. sodalis*) and gray (*M. grisescens*) bats (NWHC unpublished). The USFWS has issued a notice of petition finding and initiation of status review to list the eastern small-footed (*M. leibii*) and the northern long-eared (*M. septentrionalis*) bats in response to concern over the impact of WNS (USFWS 2011).

## Discussion

Evidence for the role of disease in the population decline to threatened or endangered status and extinction of wildlife species, and in inhibiting population recovery, has appeared to have increased in recent decades. In most of the described cases, an introduced or emerging disease or vector is the proximate cause. Identification and quantification of mortality factors at this stage can contribute to management and regulatory decisions that may enhance survival and recovery of a species.

Through diagnostic studies of >16,000 submissions of specimens from candidate, threatened, and endangered species, the NWHC has contributed importantly to the knowledge of causes of morbidity and mortality and health status in these species. NWHC data, research studies, and technical expertise have often been used in management and regulatory decisions, a few cases of which are described herein. However, the NWHC diagnostic database is comprised largely of voluntary submissions of cases of morbidity or mortality by state and federal agencies, representing a form of passive surveillance. These data thus contain numerous biases that often prevent statistically meaningful inferences of the relative importance of the various causes of mortality to a population or species as a whole. Despite this drawback, the cumulative long-term information on large numbers of a wide variety of species has been valuable to wildlife and natural resource managers. Technical assistance, consultation, and expert witness provided by wildlife disease specialists at the NWHC have also been valuable in listing decisions, the development of species recovery and implementation plans, control and management of disease outbreaks, and support of law enforcement officials in protecting listed species. Finally, applied research and development activities focused on factors limiting the recovery of listed species offer the means for new and innovative opportunities to control wildlife diseases.

The NWHC is one of a number of state, federal, and university facilities that provide diagnostic support, technical assistance, and research for wildlife diseases in North America, including those of imperiled species. For example, the Canadian Cooperative Wildlife Health Centre, the Southeastern Cooperative Wildlife Disease Study at the University of Georgia, the Wildlife Health Center at the University of California at Davis, EcoHealth Alliance, and the San Diego Zoo likewise have wildlife disease programs that benefit imperiled species in North America and beyond. As threats to imperiled species continue and are likely to increase not just from emerging diseases but other anthropogenic factors affecting their health and survival, there is a great need to better integrate and coordinate efforts to identify, understand, and manage diseases as well as other morbidity and mortality factors that are driving them to extinction or preventing their recovery. However, there is currently no national infrastructure in the U.S. to muster the various stakeholders in wildlife health to respond to wildlife health emergencies. To that end, an effort has been recently initiated by multiple state and federal agencies and organizations, including the NWHC, to establish a National Fish and Wildlife Health Network to “build a collaborative, operational framework by which government agencies, tribes, universities and professional conservation organizations cooperate to assist tribal, state and federal agencies in their responsibilities to manage these diseases.” This national network would “coordinate fish and wildlife disease surveillance, laboratory analysis, information dissemination, communication and response at a national level and across federal, state and tribal agencies and other stakeholders” (Sleeman et al. 2013).
